# Detailed Structural Analysis of the Immunoregulatory Polysaccharides from the Mycobacterium Bovis BCG

**DOI:** 10.3390/molecules27175691

**Published:** 2022-09-03

**Authors:** Lan Luo, Xuemei Song, Xiao Chang, Sheng Huang, Yunxi Zhou, Shengmei Yang, Yan Zhu, Lanyan Zhang, Yongsheng Wu, Junyin Zhang, Zhipeng Zhou, Mingyi Wu

**Affiliations:** 1 State Key Laboratory of Phytochemistry and Plant Resources in West China, Kunming Institute of Botany, Chinese Academy of Sciences, Kunming 650201, China; 2 University of Chinese Academy of Sciences, Beijing 100049, China; 3 Hunan Jiuzhitang Siqi Biological Pharmaceutical Co., Ltd., Changsha 410329, China; 4 Jiuzhitang Co., Ltd., Changsha 410205, China

**Keywords:** active ingredients, polysaccharide purification, glucan, mannan, immunomodulator

## Abstract

Bacillus Calmette-Guérin polysaccharide and nucleic acid (BCG-PSN), extracted from *Mycobacterium bovis*, is an immunoregulatory medicine commonly used in clinic. However, the structural characteristics and potential pharmacological efficacy of the polysaccharides from BCG-PSN remain unclear. Herein, two polysaccharides (BCG-1 and BCG-2) were purified and their structures were characterized. Monosaccharide composition analysis combined with methylation analysis and NMR data indicated that BCG-1 and BCG-2 were an α-D-(1→4)-mannan with (1→2)-linked branches, and an α-D-(1→4)-glucan with (1→6)-linked branches, respectively. Herein, the mannan from BCG-PSN was first reported. Bioactivity assays showed that BCG-1 and BCG-2 dose-dependently and potently increased the production of inflammatory mediators (NO, TNF-α, IL-6, IL-1β, and IL-10), as well as their mRNA expressions in RAW264.7 cells; both have similar or stronger effects compared with BCG-PSN injection. These data suggest that BCG-1 and BCG-2 are very likely the active ingredients of BCG-PSN.

## 1. Introduction

Bacillus Calmette-Guérin polysaccharide and nucleic acid (BCG-PSN) from *Mycobacterium bovis* is an immunoregulatory medicine [1]. As a strong immunomodulator, BCG-PSN can orchestrate the immune system to resist virus infections and to ameliorate autoimmune diseases [2]. BCG-PSN injection is approved by State Food and Drug Administration (China) for the prevention and treatment of chronic bronchitis, asthma and cold, and commonly used in clinic. Additionally, BCG-PSN is also effective in treating recurrent oral aphthosis [3], idiopathic urticaria [4], melanoma [5], and lichen planus [6], and it may be an effective immunoadjuvant in some immunotherapy strategies [7]. 

Plenty of research is conducted to explore the underlying mechanism of action of BCG-PSN. BCG-PSN injection can elicit the Th1-mediated immune response by facilitating T cell differentiation to the Th1 subset in vivo, as well as activate toll-like receptor (TLR) signaling pathway, and induce the secretion of Th1-type cytokines, such as IL-2 and IFN-γ [1]. In patients with chronic urticaria, BCG-PSN can promote IL-2 production by Th1 and inhibit IL-10 production by Th2; it can also decrease the release of β-hexosaminidase and regulate IgE-mediated mast cell activation [4,8]. In pulmonary tuberculosis patients, BCG-PSN can significantly enhance the phagocytic capacity of alveolar macrophage [9]. Macrophages serve as the indispensable first line of defense in the innate immune system and participate in the adaptive immune system; upon activation, they can phagocytize intracellular pathogens or promote the immune response by generating nitric oxide (NO) and inflammatory cytokines such as TNF-α, IFN-γ, IL-10, and IL-6 [10].

BCG-PSN is mainly composed of two components (polysaccharides and nucleic acids), in which the high-content and bioactive polysaccharides are macromolecules with long chains and complex chemical structures [2]. Thus, the exact structure and pharmacological characteristics of polysaccharides in BCG-PSN remain to be clarified. To date, six companies have registered for producing BCG injection under the quality criterion of Chinese Pharmacopeia. However, quality control of the injection has been challenging during preparation.

Herein, we purified the polysaccharides from BCG-PSN, characterized their chemical structures by analyzing their monosaccharide composition, methylated derivatives, and NMR data, and studied their immunomodulatory activity by testing their effects on cytokine production and mRNA expression in RAW264.7 cells. These data further contribute to the understanding of the chemical structure and bioactivity of BCG-PSN polysaccharide components, including a mannan that has not been reported, which may provide reference data for the industrial quality control and the pharmacological mechanism of BCG-PSN injection.

## 2. Materials and Methods

### 2.1. Materials

The BCG-PSN powder and BCG-PSN injection were provided by Hunan Jiuzhitang Siqi Biological Pharmaceutical Co., Ltd., China. Sephadex G-100 and Sephadex G-75 were purchased from GE Healthcare. Monosaccharides D-(+)mannose (Man), D-(-)ribose (Rib), and D-(-)arabinose (Ara) were from Alfa Aeasr (AR, Haverhill, MA, USA). D-(+)glucose (Glc), D-(+)galactose (Gal), and lipopolysaccharide (LPS) were from Sigma-Aldrich (MO, USA). Trifluoroacetic acid (TFA), 1-phenyl-3-methyl-5-pyrazolone (PMP), and α-amylase (A109181) were from Aladdin (Shanghai, China). Dulbecco’s Modified Eagle’s Medium (DMEM) culture medium was from Shanghai BasalMedia (Shanghai, China). Fetal bovine serum (FBS), streptomycin, and penicillin were from Biological Industries (Kibbutz Beit-Haemek, Israel). Cell counting kit-8 (CCK-8) was from Biosharp (Hefei, China). NO detecting kit was from Beyotime (Shanghai, China). Mouse IL-10, IL-6, and IL-1β enzyme-linked immunosorbent assay (ELISA) kits were from Multisciences (Hangzhou, China). Mouse TNF-α ELISA kit was from R&D (Minneapolis, MN, USA). E.Z.N.A. Total RNA Kit II was from Omega Bio-tek (Norcross, GA, USA). 5 × All-In-One MasterMix (with AccuRT Genomic DNA Removal Kit) and Blastaq^TM^ 2 × qPCR MasterMix were from Applied Biological Materials (Richmond, VAN, Canada). Other reagents were all commercial and of analytical grade.

### 2.2. Isolation and Purification of Polysaccharides

BCG-PSN powder (200 mg) was dissolved in distilled water; after centrifugation, the supernatant was subjected to a DEAE-52 cellulose column (3.0 cm × 13.0 cm), followed by gradient elution with sodium chloride solution (0, 0.5, 3.9 M) at a flow rate of 2 mL/min, and the eluate (10 mL/tude) was collected in sequence [11]. Then, total polysaccharides without nucleic acid were eluted by distilled water and named BCG. Alternatively, polysaccharides were also extracted from the BCG strains, which were suspended in PBS (1.0 g/mL) supplemented with Tyloxapol (0.1%). The capsular polysaccharides were extracted by vigorous shaking and centrifugation (3500× *g* 15 min), and the intracellular polysaccharides by ultrasonic cell disruption and centrifugation (3500× *g* 10 min). The supernatants were collected and filtered (0.2 μm) to remove the remaining bacterial cells. Then the filtrate was concentrated and precipitated by adding ethanol to 80% (*v*/*v*), and centrifugated (3500× *g* 15 min) to obtain the crude polysaccharides (BCG), which were dissolved in deionized water, dialyzed, and lyophilized to get the white powder.

The crude polysaccharides were further separated by Sephadex G-75 column (2 cm × 120 cm) or Sephadex G-100 column (2 cm × 120 cm), and eluted with 0.1 M NaCl (contained 0.02% NaN_3_) at a flow rate of 0.5 mL/min. The collected fractions were detected by the phenol-sulfuric acid method [12]. Based on the retention time in HPGPC, three polysaccharides (BCG-1/18.756 min, BCG-2/16.6032 min, and BCG-3/17.5032 min) were obtained, concentrated, and lyophilized. The retention time and NMR spectroscopy of the polysaccharides from BCG-PSN powder were consistent with that of polysaccharides from BCG strains. Our preliminary results showed that BCG-3 has a complicated structure. Its structural analysis requires plenty of work, thus, it will be reported alone, and the following study focuses on BCG-1 and BCG-2.

To test the presence and composition of α-glucan, BCG-PSN powder (2 g in 100 mL distilled water) was incubated with 0.8 mL α-amylase on a rotary shaker (200 rpm, pH 6.5, 70 °C) to degrade the α-glucan.

### 2.3. Homogeneity and Molecular Weight Determination

The molecular weights of BCG-1 and BCG-2 were determined by high-performance gel permeation chromatography (HPGPC) using an Agilent technologies 1260 series (Agilent, Santa Clara, CA, USA), which was equipped with RID, DAD detectors, and a Shodex OH-pak SB-804 HQ column (8 mm × 300 mm), using the eluent of 0.1 M NaCl solution (0.5 mL/min, 35 °C). The average molecular weight of BCG-1 and BCG-2 was calculated according to the standard curve of dextrans (2700, 5250, 9750, 13,050, 36,800, 64,650, 135,350 Da), using PL Cirrus GPC/SEC Software (Agilent, CA, USA), and the homogeneity was evaluated based on the HPGPC profile. Chromatographic conditions and procedures were performed according to the previous method [13].

Permethylated derivatization is a common method for in-depth analysis of glycans as it provides more mass spectra information in glycosidic linkages [14,15]. The conversion of glycans to hydrophobic derivatives such as permethylation enhances their signal strengths [14]. Thus, the polysaccharides BCG-1 and BCG-2 were permethylated using the KOH/dimethyl sulfoxide/methyl iodide for MALDI-TOF MS profiling [16,17,18].

The *M_n_* and *M_w_* were calculated from the equations [19]:

(1) $$M_{n}=\left(\sum m_{i}N_{i}\right)/\left(\sum N_{i}\right)$$

(2) $$M_{w}=\left(\sum m_{i}^{2}N_{i}\right)/\left(\sum m_{i}N_{i}\right)$$

*m_i_* and *N_i_* represent mass and intensity of the ion *i*, taking into account the proportionality among intensity of the *i*th peak, *N_i_* and the number of chains with mass *m_i_*.

### 2.4. Qualitative Analysis of Monosaccharide Composition

Monosaccharide composition of the polysaccharide was analyzed by reverse-phase HPLC after PMP derivatization [2,18,20,21]. To hydrolyze polysaccharides, 300 μL polysaccharide solution (BCG-1 or BCG-2, 1 mg/mL in deionized water) was mixed with 300 μL TFA (4 M) at 110 °C for 4 h in the sealed COD tube. Then, the reaction solution was evaporated to dryness at 70 °C to remove the residual TFA. Then, 100 μL of the hydrolyzed sample solution was incubated with 100 μL sodium hydroxide (0.6 M) and 200 μL PMP (0.5 M in methanol) at 70 °C for 60 min. After reaction, the solution pH was adjusted to 7.0 by adding HCI (0.3 M). Then, the solution was added and mixed with 2 mL of chloroform, and the chloroform layer was discarded; this step was repeated for at least six times and the top aqueous layer was collected for HPLC analysis. The standard monosaccharides (Man, Rib, Ara, Glc, and Gal) were processed by the same procedures.

The analysis of the PMP-labelled monosaccharides was carried out using an Agilent technologies 1260 series (Agilent, CA, USA) which was equipped with DAD detectors and a ZORBAX SB C18 column (4.6 mm × 150 mm, 5 μm). Mobile phase A and B (*v*/*v*, 83:17) were ammonium acetate (0.1 M, pH 5.5) and acetonitrile, respectively, at a flow rate of 1 mL/min, and UV absorbance of the effluent was monitored at 250 nm.

### 2.5. Methylation and GC-MS Analysis

Glycosidic linkage of the polysaccharides was analyzed after methylation according to the literature with minor modification [22,23]. The polysaccharide BCG-1 or BCG-2 was incubated with dimethyl sulfoxide, NaOH, and methyl iodide under the protection of nitrogen and ultrasonic conditions for 1 h. The reaction solution was added with ultrapure water to decompose methyl iodide. Then, a methylated sample was extracted with trichloromethane and evaporated to dryness. Complete hydrolysis of the methylated polysaccharides was performed by heating at 120 °C with TFA (2 M) for 4 h. The methylated sample was reduced and acetylated for GC-MS analysis. The GC-MS analysis was performed on a HP6890GC/5973 MS system (Agilent, CA, USA) equipped with an ion trap MS detector and a DB-5MS quartz capillary column (30 m × 0.25 mm, 0.25 μm). The temperature program was set as follows: the initial temperature was 80 °C, increased to 250 °C at 5 °C/min, holding for 5 min; injection temperature was 270 °C; and the ion source of the mass spectrometer was set at 230 °C. The injection volume was 1 μL and injector split ratio was 10:1.

### 2.6. UV, IR, and NMR Analysis

The UV-vis absorption spectra of the polysaccharides were recorded using a UV-2600 spectrophotometer (Shimadzu, Kyoto, Japan) in the wavelength range of 190–800 nm. The Fourier transform infrared spectroscopy (FT-IR) spectra of the polysaccharides (KBr pellets) were recorded by a Tensor-27 (Bruker, Karlsruhe, Germany) in 400–4000 cm^−1^ at room temperature.

The polysaccharide sample was dissolved in D_2_O for NMR analysis, which was performed in a Bruker Avance spectrometer of 600 or 800 MHz (Bruker, Karlsruhe, Germany), equipped with a ^13^C/^1^H dual probe in FT mode [22]. ^1^H/^1^H correlated spectroscopy (COSY), total correlation spectroscopy (TOCSY), rotating frame overhauser effect spectroscopy (ROESY), heteronuclear single-quantum correlation–total correlation spectroscopy (HSQC-TOCSY), and heteronuclear multiple bond coherence (HMBC) spectra were recorded using state-time proportion phase incrementation for quadrature detection in the indirect dimension. TMSP-2,2,3,3-D4 (D, 98%) was used as internal and external reference for the resonance measurements of BCG-1 and BCG-2, respectively.

### 2.7. In Vitro Immunomodulatory Activity Assay

#### 2.7.1. Cell Culture

The RAW 264.7 cell line from National Collection of Authenticated Cell Cultures was cultured in DMEM medium supplemented with 10% FBS (*v*/*v*), penicillin (100 U/mL), and streptomycin (100 μg/mL), under humidified conditions with 5% CO_2_ at 37 °C.

#### 2.7.2. Cell Viability Assays

Cell viability was evaluated by CCK8 assay using RAW 264.7 cells. The cells in logarithmic growth phase were adjusted to a concentration of 7.5 × 10^4^ cells/mL. Cell suspension was added to a 96-well plate and cultured at 37°C for 24 h. The cells were subsequently treated with LPS (100 ng/mL), BCG-PSN, BCG-1 or BCG-2 at different concentrations for another 24 h. The serum-free culture media was used as a control. Then, 10 μL of CCK8 solution was pipetted into each of the wells for the next four hours. Then the absorbance at 450 nm was determined by a Flexstation3 microplate reader (Molecular Device). The cell viability was calculated as following:

Cell viability (%) = A_1_/A_0_ × 100%, where A_1_ was the absorbance of treated group and the A_0_ was the absorbance of control group.

#### 2.7.3. NO and Cytokine Production

The RAW264.7 cells (4 × 10^5^ cells/mL) in logarithmic growth phase were seeded into 24-well plates (500 μL/well) and incubated for 24 h. The cells were subsequently treated with LPS (100 ng/mL), BCG-PSN injection (dilute 4 times with serum-free DMEM medium, total polysaccharides was at 87.5 µg/mL), BCG-1 or BCG-2 (1, 10, 50, and 100 μg/mL), or control (serum-free DMEM medium) for another 24 h, then the culture supernatant of cells was collected for subsequent analysis. The macrophage NO content was determined based on the Griess method [24], and the levels of TNF-α, IL-6, IL-1β, and IL-10 were detected by ELISA kits according to the manufacturer’s protocols.

#### 2.7.4. RT-qPCR Analysis

The RAW 264.7 cells with different treatments were collected and washed three times with cold PBS for RNA extraction. The total RNA was isolated using Total RNA kit II and reversed to cDNA using a 5 × All-In-One MasterMix kit (with AccuRT Genomic DNA Removal). Then, amplification of the cDNA was carried out in a total volume of 20 μL containing 10 μL Blastaq^TM^ 2 × qPCR MasterMix, 0.4 μL specific primer (10 μM), 2 μL cDNA, and 7.2 μL nuclease-free H_2_O. PCR was performed in multiple cycles using a QuantStudio 7 Flex (Thermo Scientific, Waltham, MA, USA) with the following program: denaturation at 95 °C for 3 min, annealing for 15 s, and elongation at 60 °C for 1 min. The nucleotide sequences of primers were shown in (Table S1, in the supplementary materials). β-actin gene was used as the internal reference. The expression levels of mRNA were calculated by 2^−ΔΔCt^ method.

### 2.8. Statistical Analysis

Statistical analyses were performed using GraphPad Prism 9.0 software. All the data were presented as mean ± SD. Normal distribution was determined by Shapiro-Wilk tests. Data were analyzed using one-way analysis of variance (ANOVA) followed by Dunnett’s test, *p* values less than 0.05 were considered statistically significant (* *p* < 0.05, ** *p* < 0.01, or *** *p* < 0.001).

## 3. Results

### 3.1. Isolation and Purification

**Figure 1:**
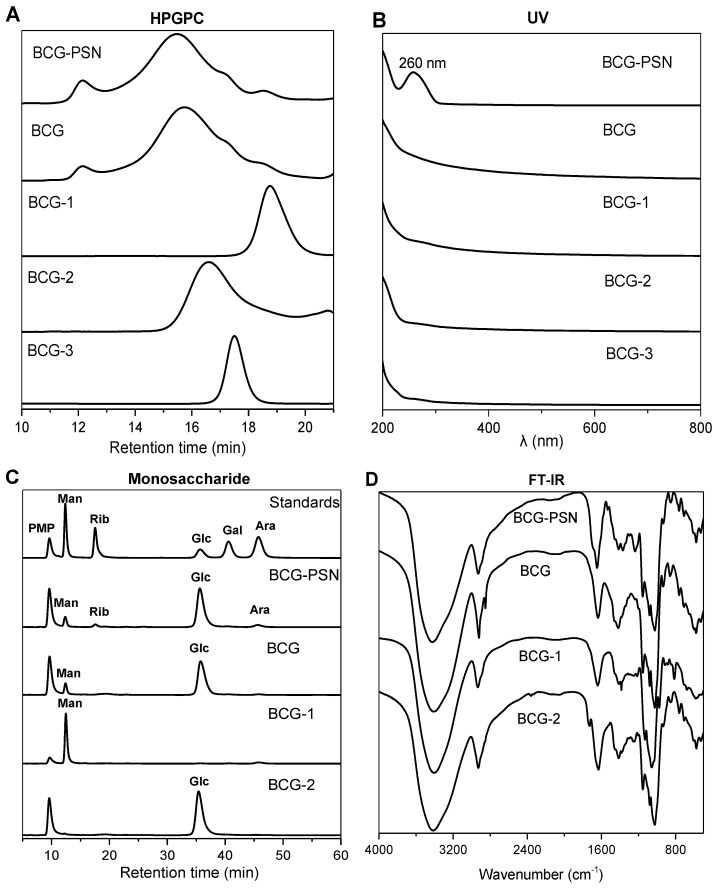

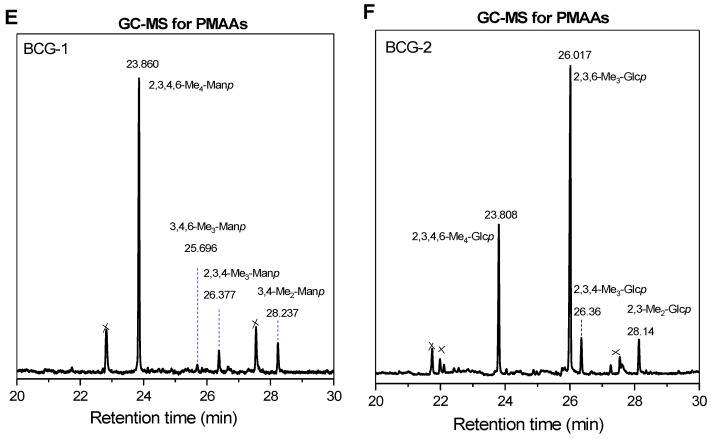
Purification and structural characterization of polysaccharides from BCG-PSN. (**A**) HPGPC profiles of BCG-PSN, BCG, BCG-1, BCG-2, and BCG-3; (**B**) UV absorption spectra of BCG-PSN, BCG, BCG-1, BCG-2, and BCG-3; (**C**) HPLC-DAD profiles of PMP derivatives of standard monosaccharides and the monosaccharides from polysaccharides; (**D**) FT-IR spectra of BCG-PSN, BCG, BCG-1, and BCG-2; (**E**) The TIC profile of PMAAs from the polysaccharide BCG-1; (**F**) The TIC profile of PMAAs from the polysaccharide BCG-2; noncarbohydrate signals are marked with ×.

In HPGPC profiles of both the BCG-PSN and total polysaccharide (BCG), a wide peak can be observed (Figure 1A and Figure S1, in the supplementary materials), suggesting that they contain distinct polysaccharides with different molecular weights. Three pure polysaccharides (BCG-1, BCG-2, and BCG-3) were further isolated from total polysaccharide BCG, their HPGPC profiles showed a single peak and symmetrical peak (Figure 1A), indicating that they are homogeneous polysaccharides. Furthermore, all the polysaccharide fractions (BCG, BCG-1, BCG-2, and BCG-3) had no absorption at 280 or 260 nm in UV spectra, indicating the absence of nucleic acids (Figure 1B). Additionally, in the RID profile of BCG-PSN, the peak in about 16 min disappeared after hydrolysis by α-amylase (Figure S1, in the supplementary materials); this indicates that BCG-PSN is rich in α-glucan as in previous reports [2,11]. The following study focused on polysaccharides BCG-1 and BCG-2 because our preliminary results, as well as the literature [25,26], showed that BCG-3 has a complicated structure (its structural analysis requires plenty of work and will be reported alone in future).

### 3.2. Molecular Weight and Monosaccharide Composition Analysis

Based on the calibration curve of standard dextrans (LogM = 11.24 − 0.501 *x* + 0.006049 *x*^2^ − 4.485e − 005*x*^3^), the weight-average molecular weights (*Mw*) of BCG-1 and BCG-2 were determined to be 4600 Da and 23,588 Da, respectively (Table S2, in the supplementary materials). Their molecular weight distributions (*Mw*/*Mn*) were 1.14 and 1.49, respectively, indicating that both polysaccharides were homogeneous.

The molecular weights of these polysaccharides were also confirmed by MALDI-TOF-MS spectrometry analysis [18,19]. In the MALDI-TOF-MS profiles of permethylated BCG-1, a mass gap between two neighboring peaks was 204.1 Da (Figure S2, in the supplementary materials). The permethylated derivative was ionized as [M + Na]^+^ ions (its relative error between experiment and theory is lower than that of [M + K]^+^ [14]), and the mass was calculated as (219.1232 (a terminal sugar) + 204.0998 (2,4,6-trimethyl glucosyl unit) × *n* + Na^+^31.0184 (OMe at reducing end residue)) Da [27] (Table S3, in the supplementary materials). The *M_n_* and *M_w_* of BCG-1 permethylated derivative were calculated to be 4158 Da and 5210 Da, respectively, the *Mw was* slightly higher than that measured by HPGPC (Table S4, in the supplementary materials). It is reported that the *Mw* upper limit of MALDI-TOF-MS spectrometry by current technology was about 5000 Da [19]. Due to the larger molecular weight of BCG-2, its signals were inadequate [15], hence further work is required to achieve optimal conditions to analyze BCG-2 [18].

Monosaccharide compositions of the polysaccharides were analyzed by reverse-phase HPLC after PMP precolumn derivatization (Figure 1C). BCG-PSN contained five monosaccharides (mannose, ribose, glucose, arabinose, and traces of galactose), among them glucose was in the highest content. Combined with the result of α-amylase hydrolysis, this indicates that BCG-PSN contains a large portion of glucan (Figure S1, in the supplementary materials). The total polysaccharide BCG was composed of mannose, glucose, and a small amount of arabinose, whereas, the purified polysaccharides BCG-1 and BCG-2 consisted exclusively of mannose and glucose, respectively (Figure 1C).

### 3.3. FT-IR Analysis

The functional groups of polysaccharides were analyzed by FT-IR spectroscopy. In the spectrum of BCG-1 (Figure 1D), the intense bands in the 3405 cm^−1^ region were ascribed to the stretching vibration of O-H, and those at 2933 cm^−1^ were produced by C-H stretching vibration [28]. The signal at 1640 cm^−1^ might be caused by the vibration of crystallized water [22,29]. A strong band between 975 and 1130 cm^−1^ was assigned to the stretching vibration of pyranose ring. The sharp peak at 814 cm^−1^ was attributed to the characteristic absorption of α-glycosidic bond of mannan [30,31], which was consistent with the results of monosaccharide composition.

Likewise, the FT-IR profile of the polysaccharide BCG-2 was also identified (Figure 1D). The signals at 3416 cm^−1^ and 2928 cm^−1^ were due to the stretching vibration of O-H and C-H, respectively. The peaks at around 1017, 1059, and 1150 cm^−1^ indicated that the monosaccharide in BCG-2 belonged to pyranose ring. Signals at 930, 840, and 760 cm^−1^ were ascribed to the characteristic absorption of α-D-glycosidic bond of glucose.

### 3.4. Methylation Analysis

For linkage analysis, methylation was conducted, and the total ion chromatograms (TIC) of the partially methylated alditol acetates (PMAAs) from BCG-1 and BCG-2 were studied. GC-MS analysis revealed that both BCG-1 and BCG-2 derivatives contained four PMAAs (Figure 1E,F). The data on glycosidic linkage and molar ratio were summarized in Table 1. According to the ESI-MS information (Figure S3, in the supplementary materials) and the GC-EIMS databases of PMAAs (Complex Carbohydrate Research Center, University of Georgia), the peaks in TIC of BCG-1 were identified as 1,5-di-*O*-acetyl-2,3,4,6-tetra-*O*- methyl-D-mannitol (2,3,4,6-Me_4_-Man), 1,2,5-tri-*O*-acetyl-3,4,6-tri-O-methyl-D-mannitol (3,4,6-Me_3_-Man), 1, 5,6-tri-*O*-acetyl-2,3,4-tri-*O*-methyl-D-mannitol (2,3,4-Me_3_-Man), and 1,2,5,6-tri-*O*-acetyl-3,4-di-*O*-methyl-D-mannitol (3,4-Me_2_-Man), with the molar ratio of 35.86:1.00:3.10:1.52 based on the peak areas (Table 1). The results indicated the presence of terminal 2-, 6-, 2,6-linked Man*p* pyranoside residues.

Similarly, the PMAAs of BCG-2 were identified as 1,5-di-*O*-acetyl-2,3,4,6-tetra-*O*-methyl-D-glucitol (2,3,4,6-Me_4_-Glc), 1,4,5-tri-*O*-acetyl-2,3,6-tri-*O*-methyl-D-glucitol (2,3,6-Me_3_-Glc), 1,5,6-tri-*O*-acetyl- 2,3,4-tri-*O*-methyl-D-glucitol (2,3,4-Me_3_-Glc), and 1,4,5,6-tri-*O*-acetyl-2,3-di-*O*-methyl-D-glucitol (2,3-Me_2_-Glc), with the molar ratio of 5.16:10.38:1.13:1.00 (Figure 1F, Table 1, and Figure S4, in the supplementary materials). These results indicated the presence of terminal 4-,6-, 4,6-linked Glc*p* residues.

### 3.5. NMR Analysis

**Figure 2:**
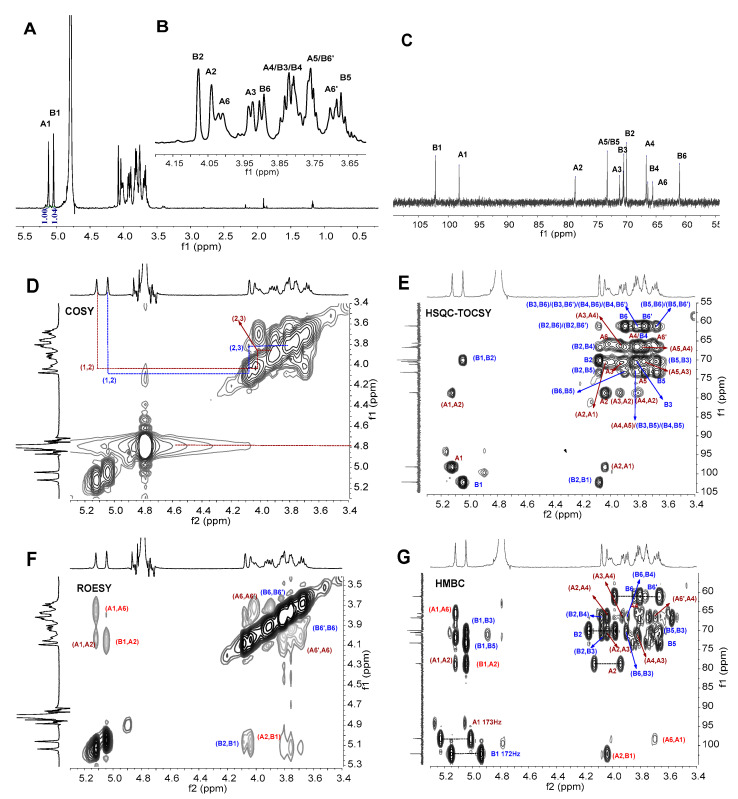
^1^H (**A**,**B**), ^13^C (**C**), COSY (**D**), HSQC-TOCSY (**E**), ROESY (**F**), HMBC (**G**) NMR spectra of BCG-1. Chemical shifts are relative to internal trimethylsilylpropionic acid sodium at 0 ppm. The letters A (maroon) and B (blue) represented 2,6-*O*-α-Man*p* (Residue A) and t-α-Man*p* (Residue B), respectively.

**Figure 3:**
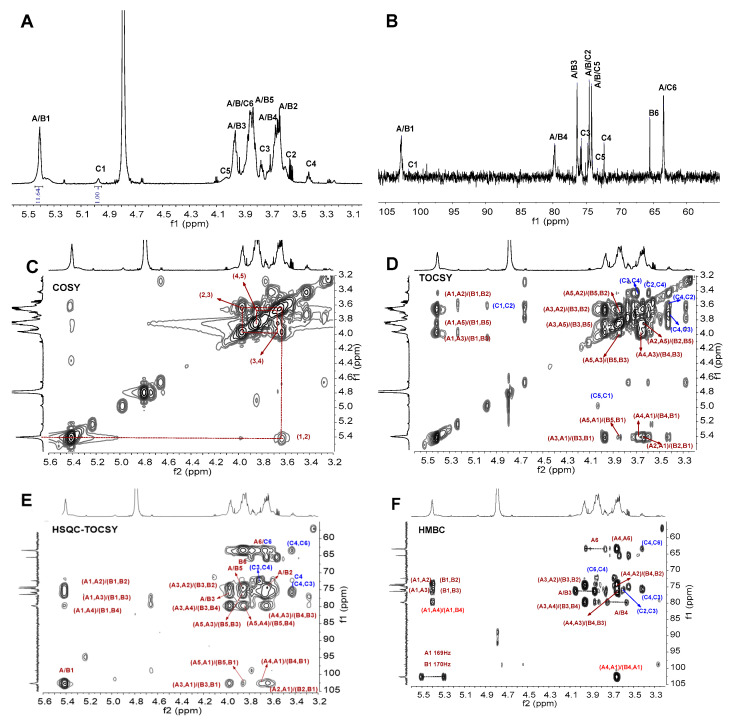
^1^H (**A**), ^13^C (**B**), COSY (**C**), TOCSY (**D**), HSQC-TOCSY (**E**), HMBC (**F**) NMR spectra of BCG-2. Chemical shifts are relative to external trimethylsilylpropionic acid sodium at 0 ppm. The letters A/B (maroon) represented →4)-α-Glc→1 (residue A)/→4,6)-α-Glc(1→ (residue B). The letters C (blue) represented t-α-Glc (1→ (residue C).

The structural features of BCG-1 and BCG-2 were further elucidated by ^1^H and ^13^C NMR analysis. Their chemical shifts were assigned according to 1D (^1^H, ^13^C) and 2D COSY, ROESY, HSQC-TOCSY, and HMBC data (Figure 2 and Figure 3 and Table 2).

**Figure 4:**
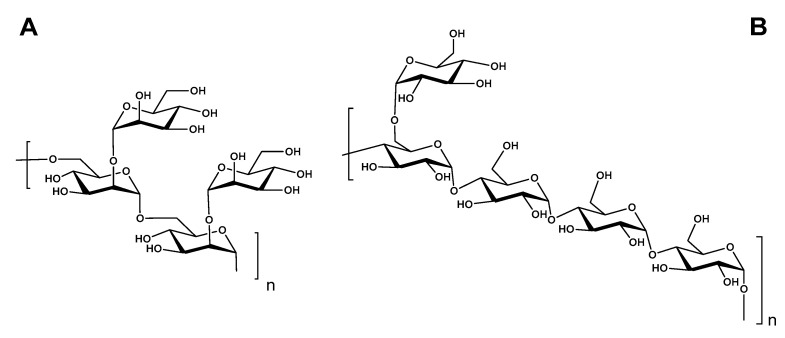
Proposed structures of BCG-1 (**A**) and BCG-2 (**B**) purified from BCG-PSN.

In the ^1^H NMR spectrum of BCG-1, an anomeric zone (δ_H_ 4.9–5.5 ppm) exhibited two signals at 5.12 and 5.04 ppm (Figure 2A,B), which were unambiguously assigned as the anomeric proton resonance of residue A and B, respectively. The molar proportion of A and B residues was about 1:1.04 according to the signal integration. The ^13^C NMR spectrum also displayed two signals in the anomeric region at 98.2 ppm and 102.2 ppm (Figure 2C), which were correlated with anomeric protons [31]. We thus deduced that BCG-1 may contain two mannosyl units, 2,6-*O*-α- Man*p* (Residue A), and t-α-D-Man*p* (Residue B). The detailed proton signals from H-2 to H-6 of the two residues were assigned using the ^1^H-^1^H COSY (Figure 2D) and HSQC-TOCSY (Figure 2E) data. The chemical shifts from δ_H_ 3.3 ppm to δ_H_ 4.0 ppm, showing overlapping peaks, were assigned to the protons of C-2 to C-6 in glycosidic rings [32,33]. For residue A, the typical signals of C-2 at ~78.6 ppm were shifted ~6 ppm to low-field of δ_C-2_ for α-mannose, and the H-2 signals at ~4.04 ppm were shifted ~0.2 ppm to δ_H-2_ for α-mannose, indicating that residue A was *O*-substituted at 2 position. In addition, the C-6 signals at ~65.6 ppm for residue A were shifted ~4.6 ppm to low-field of δ_C-6_, supporting the high substitution at C-6 of residue A. Combining the results of methylation analysis (Table 1) and NMR data, it suggested that BCG-1 has (1→2) and (1→6)-linkages. The linkages of the glycosyl residues were further confirmed by 2D ROESY (Figure 2F) and HMBC analysis (Figure 2G). The H-1 of residue A had a strong inter-residue ROE connected to H-6 of adjacent residue in addition to intra-residue ROE, indicating that residue A was linked to C-6 of adjacent residue. Similarly, the H-1 of residue B had a strong inter-residue ROE with H-2 of residue A, indicating that residue B was linked to C-2 of residue A. In addition, the HMBC spectrum (Figure 2G) also showed the sequence-defining C-1 of residue A and H-6 of adjacent residue. Accordingly, BCG-1 should have the linear backbone of →6-A-1→6-A-1→ and the side chain of B-1→2-A. To further confirm the configurations at the glycosidic linkages, the direct coupling constants (^1^*J*_C–H_) of C-1 of each saccharide from the HMBC spectrum were analyzed (Figure 2G). The large values of ~170 Hz for these mannose residues indicated that the protons at C-1 were equatorial [34], therefore the configurations of C-1 were determined to be α-D-mannose. This was consistent with the strongly specific rotation (+84°) and proton shifts of anomeric signals of mannan residue (Table 2). Taken together, the structure of BCG-1 was proposed in Figure 4A.

For BCG-2, its chemical shifts in 1D and 2D NMR spectra were also assigned (Table 2 and Figure 3). In the ^1^H NMR spectrum (Figure 3A), the chemical shifts at 5.41 ppm, 5.39 ppm, and 4.96 ppm were anomeric proton signals of Residues A, B, and C, respectively [35], which corresponded to two low-field signals at 102.6 and 101.6 ppm in its ^13^C spectrum (Figure 3B). The molar ratio of A and B to C was about 4:1, according to the integral area. The signals at δ_H_ 3.4–4.1 ppm were assigned to protons in other locations of saccharide rings [11,22,35,36]. Based on COSY and TOCSY spectra (Figure 3C,D), the chemical shifts of H2~H6 were in 3.63, 3.96, 3.66, 3.86, 3.88, and 3.76 ppm, respectively. The carbon signals were assigned based on the assignment of the protons in HSQC spectrum. The HSQC-TOCSY and HMBC spectra clearly showed the related signals between C-H on the saccharide ring (Figure 3E,F). In the ^13^C NMR spectrum (Figure 3B), the downfield signals at 102.6 ppm and 101.6 ppm were attributed to anomeric carbons of A/B and C, respectively. The typical C-4 signals at ∼79.8 ppm for A and B residues were shifted ~8 ppm to low-field of δ_C-4_, demonstrating that A and B residues were substituted at 4-position [22,35,37]. The signals of *O*-substituted C-6 and unsubstituted C-6 were at 65.4 ppm and 63.4 ppm, respectively [22]. Furthermore, the methylation analysis (Table 1) and HMBC spectrum (Figure 2G) indicated that BCG-2 has the (1→4) linked glucan main chain and (1→6)-linked branches (Figure 4B). The branching point presented on the average of every tetrasaccharide fragment, indicating a high branching degree of the α-glucan. The structure proposed for BCG-2 was similar to that of the glucans from *M. bovis* [2,11] and *M. tuberculosis* [38], while BCG-2 exhibited different saccharide sequences from these reported glucans.

### 3.6. Effects of BCG-1 and BCG-2 on the NO Stimulation

**Figure 5:**
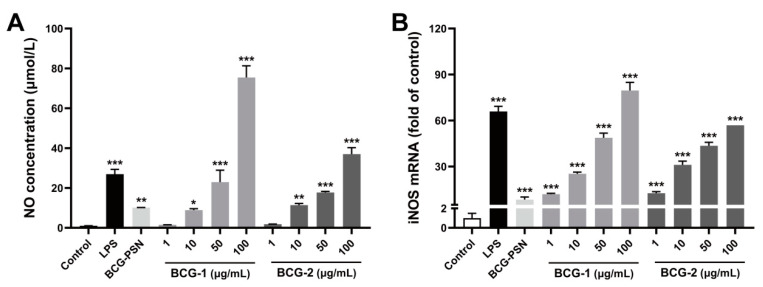
Effects of LPS, BCG-PSN, BCG-1, and BCG-2 on the production of NO (**A**) and mRNA expressions of *iNOS* (**B**) in RAW264.7 cells. LPS was at 100 ng/mL, BCG-PSN was at 87.5 µg/mL. * *p* < 0.05, ** *p* < 0.01, and *** *p* < 0.001 vs. control, one-way ANOVA, Dunnett’s multiple comparisons test.

To study the immunomodulatory activity of polysaccharides BCG-1 and BCG-2, their effects on NO production and inducible nitric oxide synthase (iNOS) expression were tested using RAW264.7 cells. NO is known as a signaling molecule released by activated macrophages, which is critical to defense against microbe invasion and tumor cells [39]. Additionally, iNOS is a crucial enzyme responsible for NO generation [40]. After stimulation with LPS or BCG-PSN injection, RAW264.7 cells significantly increased NO secretion, compared with control (Figure 5A). Both BCG-1 and BCG-2 dose-dependently increased NO production by macrophages, at 10 µg/mL or above their effects were significant, and at 50 µg/mL or above stronger than BCG-PSN. Consistently, BCG-1 and BCG-2 at 1~100 µg/mL also potently enhanced the mRNA expression of iNOS (Figure 5B). These results suggested that BCG-1 and BCG-2 can enhance NO production from macrophages.

### 3.7. Effects of BCG-1 and BCG-2 on the Production of Cytokines

**Figure 6:**
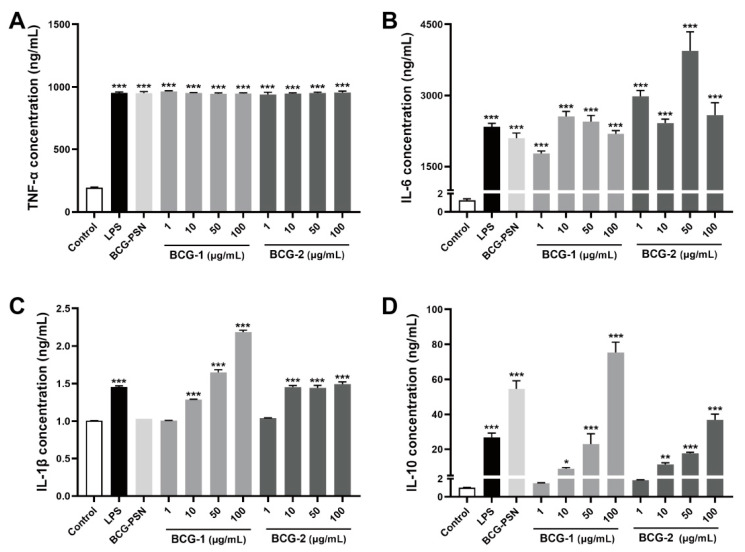
Effects of LPS, BCG-PSN, BCG-1, and BCG-2 on cytokines TNF-α (**A**), IL-6 (**B**), IL-1β (**C**), IL-10 (**D**) produced by the RAW 264.7 cells. LPS was at 100 ng/mL, BCG-PSN was at 87.5 µg/mL. * *p* < 0.05, ** *p* < 0.01, and *** *p* < 0.001 vs. control, one-way ANOVA, Dunnett’s multiple comparisons test.

Activated macrophages can release inflammation cytokines, such as TNF-α, IL-6, IL-1β, and IL-10 [41]. In turn, the levels of these cytokines can be used to evaluate the activation degree of macrophage. At the tested concentration, BCG-1, BCG-2, and BCG-PSN injection could potently promote the release of TNF-α from RAW264.7 cells (Figure 6A), BCG-1 and BCG-2 at 1 μg/mL increased TNF-α to more than four folds of the control, comparable to the effect of LPS (100 ng/mL), and the production of TNF-α seemed to plateau without further increase with higher doses. BCG-1, BCG-2, and BCG-PSN injection also significantly increased the production of IL-6 to more than 1000 folds of the control, with the activity comparable to LPS (100 ng/mL) (Figure 6B). Although the activity of BCG-1 and BCG-2 in promoting IL-1β secretion was much weaker (exhibiting only significant effects at 10 μg/mL or above), it is also comparable to the potency of LPS (Figure 6C). Our results showed that BCG-1 and BCG-2 can also stimulate the production of IL-10 in a dose-dependent manner (Figure 6D). These results are consistent with previous reports of the immunomodulatory effects of polysaccharides [42,43,44]. TNF-α, IL-6, IL-1β, and IL-10 are essential immunomodulatory cytokines and play critical roles in innate and adaptive immune responses [45,46]. The results suggested that BCG-1 and BCG-2 exhibit potent immunomodulatory effects, comparable to or stronger than BCG-PSN, indicating that they may be the major active ingredients of BCG-PSN injection.

### 3.8. Effects of BCG-1 and BCG-2 on the Expression of Inflammation Genes

**Figure 7:**
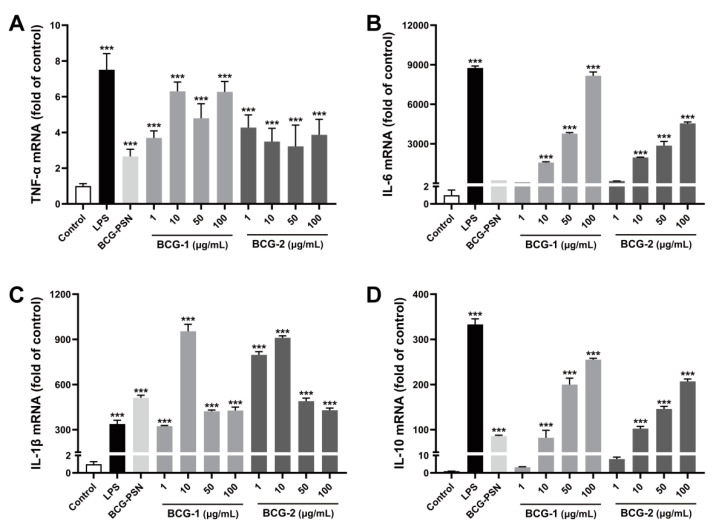
Effects of LPS, BCG-PSN, BCG-1, and BCG-2 on the mRNA expressions of TNF-α (**A**), IL-6 (**B**), IL-1β (**C**), IL-10 (**D**) in the RAW 264.7 cells. LPS was at 100 ng/mL, BCG-PSN was at 87.5 µg/mL. *** *p* < 0.001 vs. control, one-way ANOVA, Dunnett’s multiple comparisons test.

Since cytokine productions are related to their gene expressions, we next examined the effects of BCG-1 and BCG-2 on their relevant mRNA expression using RT-qPCR tests (Figure 7). Obviously, untreated RAW264.7 cells expressed low mRNA levels of all inflammatory cytokines (TNF-α, IL-6, IL-1β, and IL-10). After stimulation with BCG-PSN injection, the mRNA expression of cytokines TNF-α, IL-1β, and IL10 significantly increased except for IL-6. BCG-1 and BCG-2 at 1 μg/mL or above markedly up-regulated the expression levels of TNF-α and IL-1β, while it required 10 μg/mL or above to significantly increase IL-6 and IL-10. Taken together, it suggested that BCG-1 and BCG-2 increased the production of inflammation cytokines from RAW264.7 cells, at least partly by enhancing their expression at the transcriptional level.

In summary, the purified polysaccharides BCG-1 and BCG-2 showed obvious immunomodulatory effects by promoting the production of NO, TNF-α, IL-6, IL-1β, and IL-10 from macrophages, similar to BCG-PSN injection.

## 4. Discussion

Clinical trials have demonstrated that BCG-PSN is effective in the treatment of asthma, atopic dermatitis, chronic urticaria, oral, and cutaneous lichen planus [1,47]. The polysaccharide components are believed to be the active ingredients of BCG-PSN injection. Our present study indicated that BCG-PSN was composed of three polysaccharides, mostly α-D-glucan, a small amount of α-D-mannan, and arabinomannan. The chemical structures of two purified polysaccharides, BCG-1 and BCG-2, from BCG-PSN was characterized. The polysaccharide BCG-1 was an α-D-(1→4) mannan with (1→2)-linked branches, and BCG-2 was a glucan with α-D-(1→4)-linked backbone and (1→6)-linked branches.

In the past decades, lipomannan (LM), arabinomannan (AM), and mannosylated lipoarabinomannan (ManLAM) from *M. bovis* BCG were reported [25,26], while the structure–function relationship of these polysaccharides from BCG-PSN injection was less studied. A polysaccharide BDP and a water-soluble glucan (BCG-PASW) from BCG-PSN were reported [2,11], but their biological activity remains unclear. The present study has extended our knowledge in the structural diversity and immunostimulatory capability of polysaccharides from *Mycobacterium* species. Moreover, our results have important implications both for the understanding of active ingredients and quality control of BCG-PSN.

Macrophage activation is primarily regulated by recognizing pathogens through pattern recognition receptors such as Toll-like receptors (TLRs) [39,48]. TLRs could induce MyD88- and TRIF- dependent pathways, resulting in the activation of transcription factors NF-κB (nuclear factor kappa-B) and IRF3 (interferon regulatory factor 3), and the up-regulation of co-stimulatory markers and proinflammatory gene expression such as iNOS, TNF-α, IL-6, IL-1, and IL-8 [48,49]. Furthermore, TLR-MyD88 pathway is involved in dendritic cell maturation, thereby bridging the innate and adaptive immune response [50]. It is reported that BCG-PSN can enhance adaptive immunity via activating TLR signaling pathways and inducing the secretion of proinflammatory cytokines [1]. In our study, macrophages stimulated by BCG-1, BCG-2, or BCG-PSN injection increased the gene expression of proinflammatory cytokines TNF-α, IL-6, IL-1β, as well as inflammatory mediator iNOS (Figure 7). Consequently, these polysaccharides all substantially increased proinflammatory cytokines (NO, TNF-α, IL-6, and IL-1β) produced by macrophages (Figure 6). Therefore, the active ingredients in BCG-PSN, such as BCG-1 and BCG-2, may strengthen the immune responses, owing to activating the TLR pathway and inducing the production of inflammatory cytokines.

Additionally, BCG-PSN can induce macrophages to kill bladder cancer cells, possibly by expression of and synergy with Th1-stimulating cytokines [51,52]. IL-10 is a Th2 cytokine with anti-inflammatory and immunosuppressive properties; it may prevent the secretion of proinflammatory cytokines (TNF-α and IL-1β) and NO by macrophages [53,54]. Some studies have suggested that blockade of IL-10 may improve the therapeutic efficacy of the BCG vaccine in the treatment of bladder cancer patients [55,56]. Our in vitro data showed that although BCG-1 and BCG-2 increased IL-10 production, their activities were weaker than the positive control LPS. It is suggested that the polysaccharide chondroitin sulfate may possess both pro-inflammatory and anti-inflammatory effects [44]. However, the exact in vivo effect of these polysaccharides requires further study.

Taken together, BCG-1 and BCG-2 are most likely the functional components of BCG-PSN. However, given the difference of isolation, purification, and structural characterization, there may be other active ingredients that have not been purified and identified in BCG-PSN. For instance, in our study, we obtained another polysaccharide (arabinomannan, BCG-3), but its complex structure remains to be clarified in our future work. Since the bioactivity of polysaccharides depends on their structural features, different polysaccharide components or proportions may impact the immunomodulatory activity of BCG-PNS. Thus, it is essential to elucidate the structure–activity relationship of polysaccharides in BCG-PSN.

## 5. Conclusions

In conclusion, two polysaccharides, BCG-1 and BCG-2, were obtained from BCG-PSN. Chemical characteristic analysis revealed that BCG-1 was an α-D-(1→4) mannan with (1→2)-linked branches, and BCG-2 was an α-D-(1→4) glucan with (1→6)-linked branches. Both polysaccharides showed potent immunomodulatory effects, similar to BGC-PSN injection, significantly inducing the production of NO, TNF-α, IL-6, IL-1β, and IL-10, and their mRNA expression by RAW 264.7 cells. These results suggested that BCG-1 and BCG-2 were most likely the functional ingredients of BCG-PSN injection.

## Figures and Tables

**Table 1:** GC-MS of alditol acetate derivatives of the BCG-1 and BCG-2 methylated products.

PMAA Derivatives ^a^	Type of Linkage	Relative Retention Time ^b^	Molar Ratio	Mass Fragments (m/z)
BCG-1				
2,3,4,6-Me_4_- Man*p*	t-Man*p*	1.00	35.86	71, 87, 101, 129, 145, 162, 205
3,4,6-Me_3_-Man*p*	2- Man*p*	1.08	1.00	87, 129, 161, 189
2,3,4-Me_3_-Man*p*	6- Man*p*	1.11	3.10	71, 87, 101, 118, 129, 161, 189
3,4-Me_2_- Man*p*	2,6- Man*p*	1.18	3.52	87, 99, 129, 189
BCG-2				
2,3,4,6-Me_4_-Glc*p*	t-Glc*p*	1.00	5.16	87, 102, 118, 129, 145, 162, 205
2,3,6- Me_3_- Glc*p*	4- Glc*p*	1.09	10.38	87, 100, 118, 129, 233
2,3,4-Me_3_- Glc*p*	6- Glc*p*	1.17	1.13	87, 100, 118, 129, 161, 189
2,3-Me_2_- Glc*p*	4,6- Glc*p*	1.18	1.00	100, 117, 129, 261

^a^ 2,3,4,6-Me_4_-Man = 1,5-di-*O*-acetyl-2,3,4,6-tetra-*O*-methyl-mannose, etc. ^b^ Relative retention times of the corresponding alditol acetate derivatives compared with 1,5-di-*O*-acetyl-2,3,4,6-tetra-*O*-methyl-D-mannitol (for BCG-1) and 1,5-di-*O*-acetyl-2,3,4,6-tetra-*O*-methyl-D-glucitol (for BCG-2).

**Table 2:** ^1^H and ^13^C NMR chemical shifts of the BCG-1 and BCG-2 in D_2_O.

Sugar Residues	Chemical Shifts (ppm)							
		1	2	3	4	5	6a	6b
BCG-1								
2,6-*O*-α-Man*p* (A)	H	5.12	4.04	3.94	3.82	3.77	4.01	3.68
	C	98.2	78.6	70.5	66.4	73.1	65.6	-
t-α-Man (B)	H	5.04	4.08	3.82	3.82	3.68	3.90	3.76
	C	102.2	70.0	70.6	66.4	72.8	61.0	-
BCG-2								
→4)-α-Glc→1 (A)	H	5.41	3.63	3.96	3.66	3.86	3.88	3.76
	C	102.6	74.5	76.3	79.8	74.1	63.4	-
→4,6)-α-Glc(1→ (B)	H	5.39	3.63	3.96	3.66	3.83	3.86	3.85
	C	102.6	74.5	76.3	79.8	74.1	65.5	-
t-α-Glc(1→ (C)	H	4.96	3.61	3.71	3.41	4.04	3.85	
	C	101.6	74.5	75.7	72.3	73.4	63.4	

## Data Availability

Not applicable.

## Supplementary Materials

The following supporting information can be downloaded at: https://www.mdpi.com/article/10.3390/molecules27175691/s1 Figure S1: HPGPC profiles of BCG-PSN before (A) and after (B) α-amylase hydrolysis, Figure S2: MALDI-TOF MS spectrum of BCG-1, Figure S3: MS fragments of PMAAs of BCG-1 and its deduced residues, Figure S4: MS fragments of PMAAs of BCG-2 and its deduced residues, Figure S5: Effects of BCG-PSN, BCG-1 and BCG-2 on the cell viability, Table S1: Primer sequences for RT-qPCR analysis, Table S2: Molecular weights of two polysaccharides, Table S3: MALDI-TOF MS data of BCG-1, Table S4: Weight average (Mw), number average (Mn) and polydispersity index (PI) of BCG-1 determined by HPGPC and MALDI-TOF MS.

## Abbreviations

BCG-PSN: Bacillus Calmette-Guérin polysaccharide and nucleic acid; COSY, correlated spectroscopy; DMEM, dulbecco’s modified eagle’s medium; ESI-MS, electrospray ionization mass spectrometry; FTIR, fourier-transform infrared spectroscopy; GC-MS, gas chromatograph-mass spectrometer; HMBC, heteronuclear multiple bond coherence; HPGPC, high-performance gel permeation chromatography; HPLC, high performance liquid chromatography; HSQC–TOCSY, heteronuclear single-quantum correlation–total correlation spectroscopy; IL, interleukin; IR, infrared; iNOS, Inducible nitric oxide synthase; LPS, lipopolysaccharide; MALDI-TOF MS, matrix-assisted laser desorption/ionization time of flight mass spectrometry; Mn, number-average molecular weight; Mw, weight-average molecular weight; MyD88, myeloid differentiation factor 88; NMR, nuclear magnetic resonance; PMAAs, partially methylated alditol acetates; PMP, 1-phenyl-3-methyl-5-pyrazolone; qPCR, quantitative PCR; ROESY, rotating frame overhauser effect spectroscopy; RT-PCR, real-time polymerase chain reaction; TFA, trifluoroacetic acid; TIC, total ion chromatograms; TLR, toll-like receptor; TNF-α, tumor necrosis factor-α; TOCSY, total correlation spectroscopy.
