# Supplementary material for: Detailed Structural Analysis of the Immunoregulatory Polysaccharides from the Mycobacterium Bovis BCG
Source: Molecules. 2022 Sep 3;27(17):5691. doi: 10.3390/molecules27175691 (PMC9458083; doi:10.3390/molecules27175691)
Supplement: Supplementary file 1 [file molecules-27-05691-s001.zip › molecules-1879493-Supplementary.pdf]

Supplementary data

Detailed structural analysis of the immunoregulatory polysaccharides from the *Mycobacterium bovis* BCG

Lan Luo [1, †], Xuemei Song [1, 2, †], Xiao Chang [1, †], Sheng Huang [3, 4], Yunxi Zhou [3], Shengmei Yang [3], Yan Zhu [3], Lanyan Zhang [3], Yongsheng Wu [3, 4], Junyin Zhang [1], Zhipeng Zhou [1], Mingyi Wu [1, 2, *]

Contents:

Figure S1. HPGPC profiles of BCG-PSN before and after α-amylase hydrolysis.

Figure S2. MALDI-TOF MS **spectrum** of BCG-1.

Figure S3. MS fragments of PMAAs of BCG-1 and its deduced residues.

Figure S4. MS fragments of PMAAs of BCG-2 and its deduced residues.

Figure S5. Effects of BCG-PSN, BCG-1 and BCG-2 on the cell viability.

Table S1. Primer sequences for RT-qPCR analysis.

Table S2. Molecular weights of two polysaccharides.

Table S3. MALDI-TOF MS data of BCG-1.

Table S4. Weight average (*Mw*), number average (*Mn*) and polydispersity index (PI) of BCG-1 determined by HPGPC and MALDI-TOF MS.

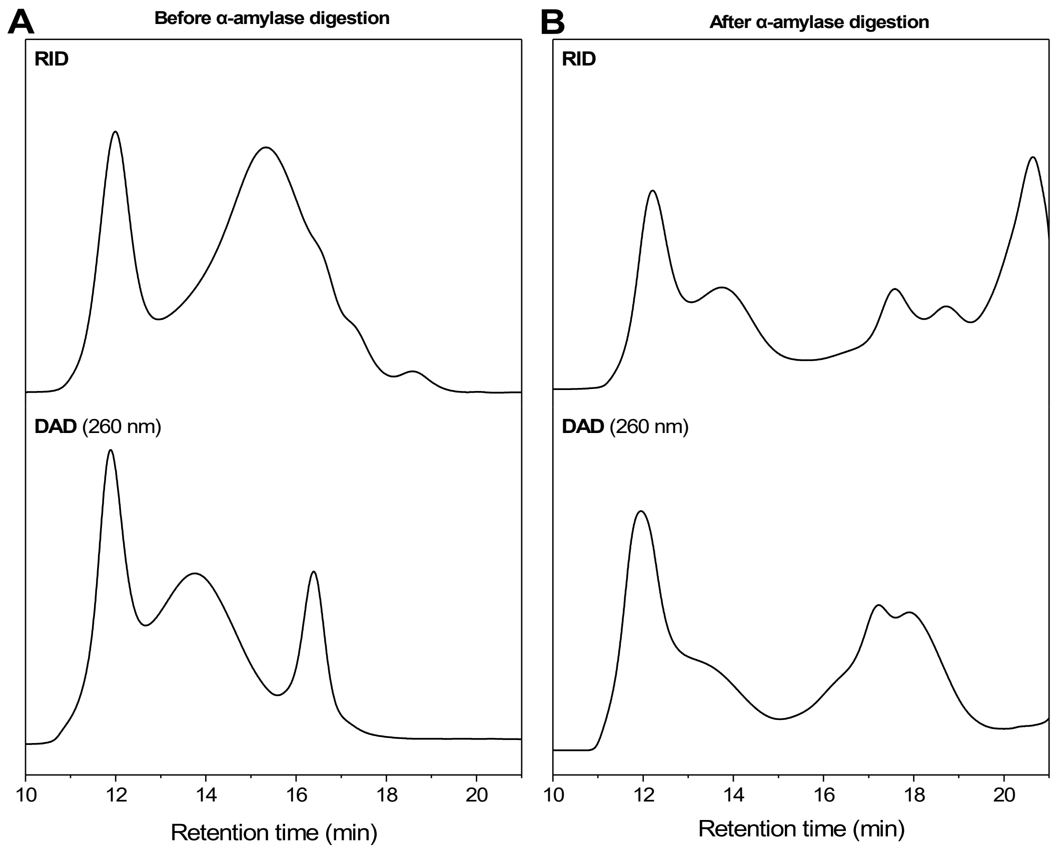

Figure S1. HPGPC profiles of BCG-PSN before (A) and after (B) α-amylase hydrolysis.

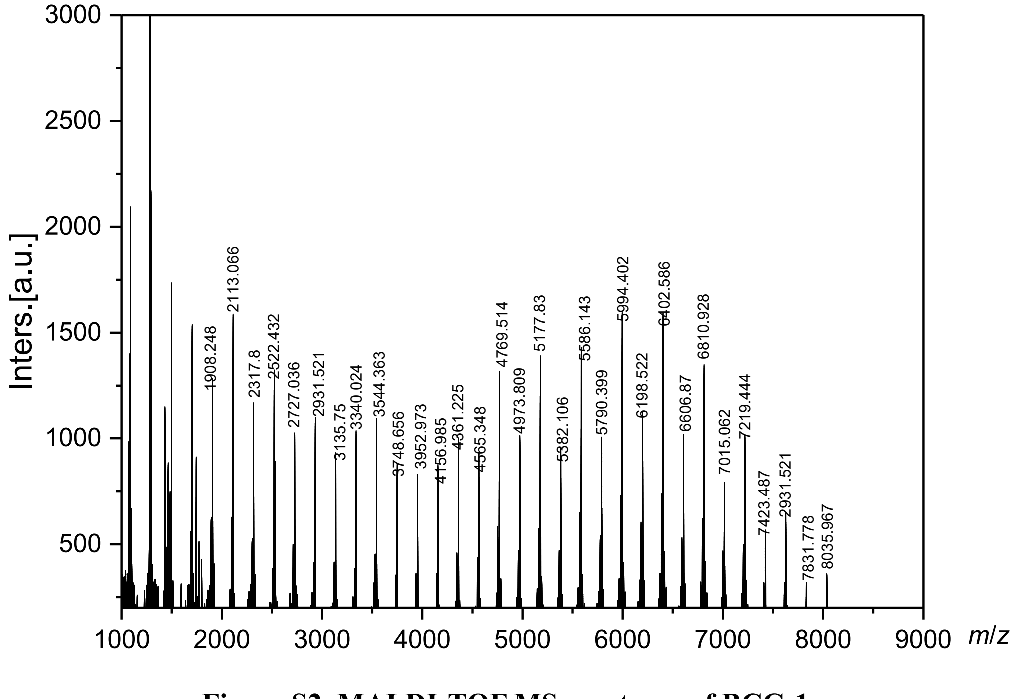

Figure S2. MALDI-TOF MS spectrum of BCG-1

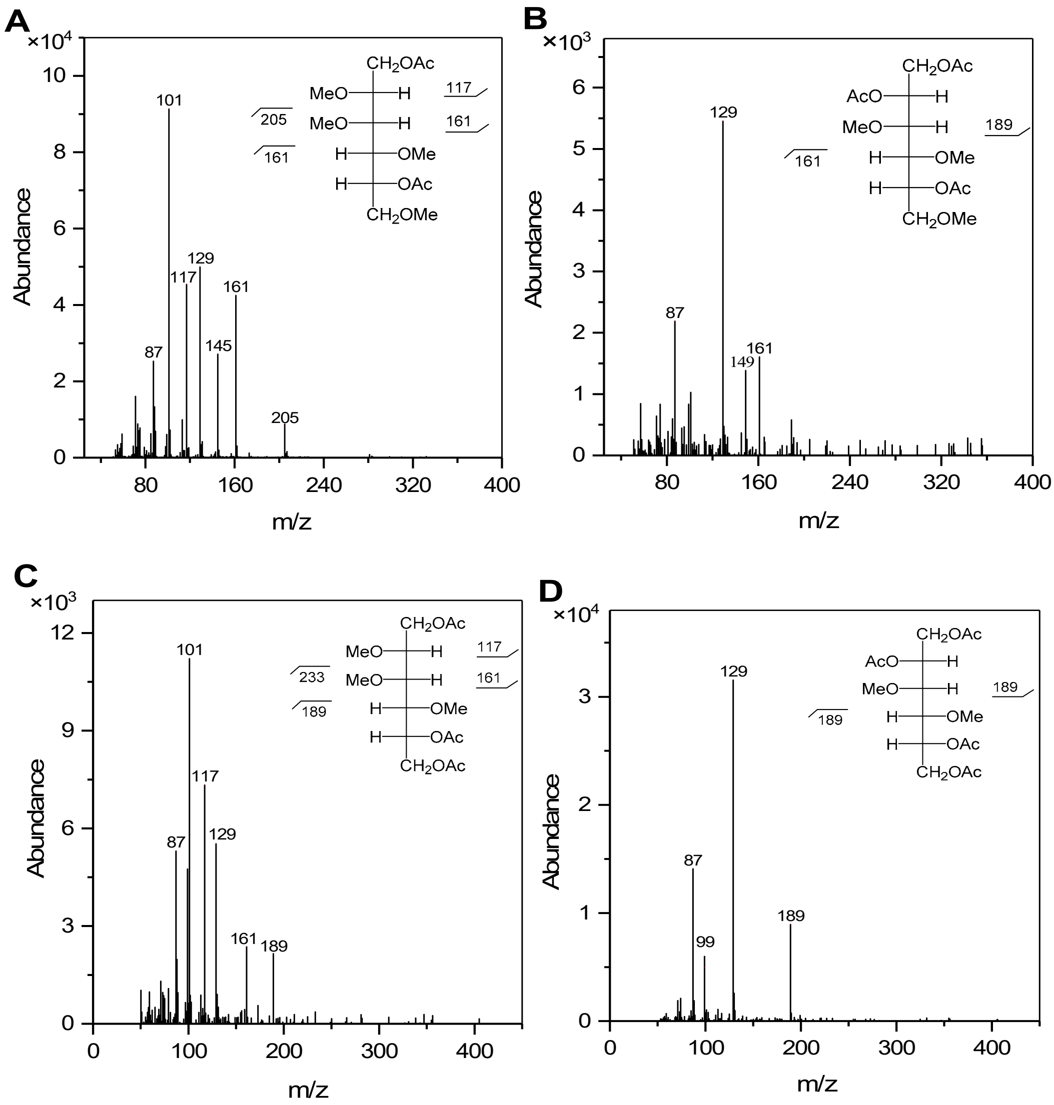

Figure S3. MS fragments of PMAAs of BCG-1 and its deduced residues.

The mass profile of 1,5-di-O-acetyl-2,3,4,6-tetra-O-methyl-D-mannitol (**A**), 1,2,5-tri-O-acetyl-3,4,6-tri-O-methyl-D-mannitol (**B**) 1,5,6-tri-O-acetyl-2,3,4-tri-O-methyl-D-mannitol (**C**) and 1,2,5,6- tetra-O-acetyl-3,4-di-O-methyl-D-mannitol (**D**).

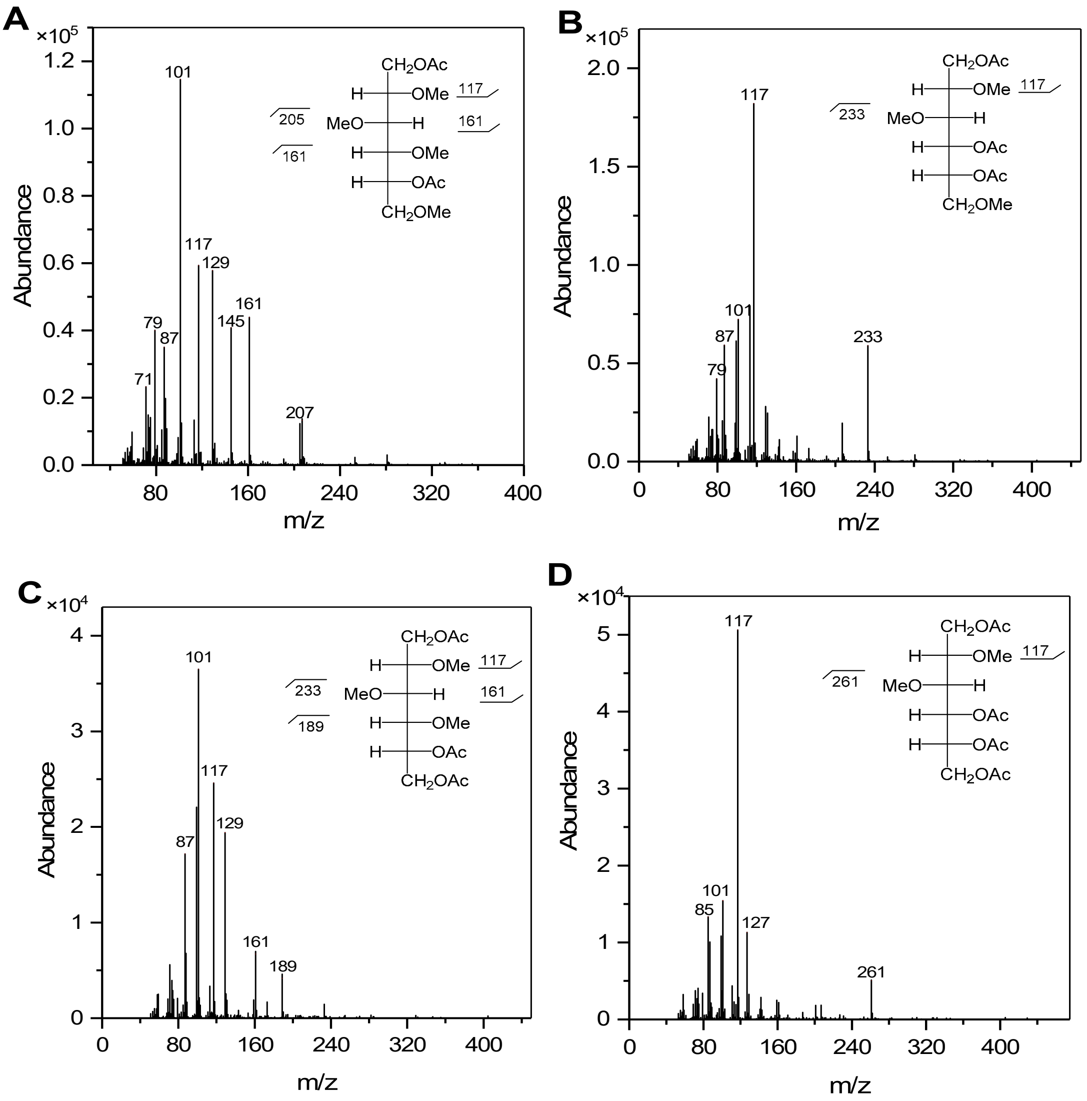

Figure S4. MS fragments of PMAAs of BCG-2 and its deduced residues.

The mass profile of 1,5-di-O-acetyl-2,3,4,6-tetra-O-methyl-D-glucitol (**A**), 1,4,5-tri-O-acetyl-2,3,6-tri-O-methyl-D-glucitol (**B**); 1,5,6-tri-O-acetyl-2,3,4-tri-O-methyl-D-glucitol (**C**) and 1,4,5,6-tetra -O-acetyl-2,3-di-O-methyl-D-glucitol (**D**).

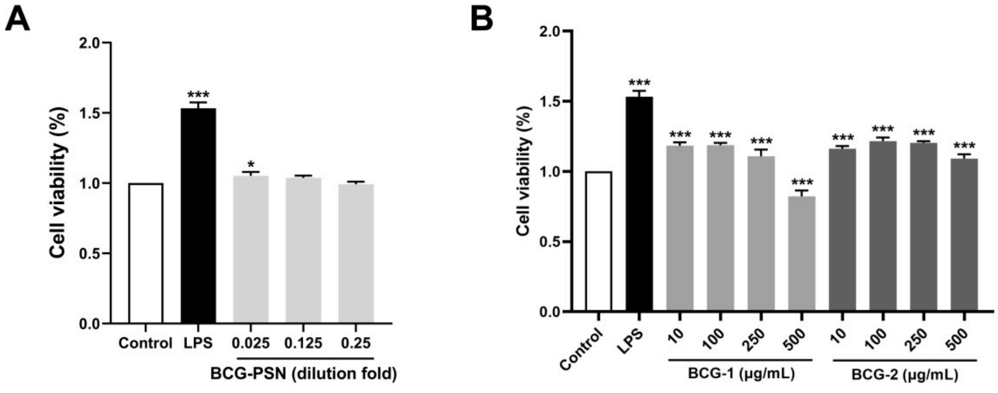

Figure S5. Effects of BCG-PSN (A), BCG-1 and BCG-2 (B) on the cell viability.

LPS was at 100 ng/mL. Mean ± SD, n = 5, $^*P < 0.05$, $^{**}P < 0.01$ and $^{***}P < 0.001$ *vs*. control, one-way ANOVA, Dunnett's multiple comparisons test.

Table S1. Primer sequences for RT-qPCR analysis.

Genes	Forwards (5’-3’)	Reverses (5’-3’)
Atcb	CCACAGCTGAGAGGGAAATC	AAGGAAGGCTGGAAAAGAG
Tnfa	GACGTGGAACTGGCAGAAGAG	TTGGTGGTTTGTGAGTGTGAG
Il6	AGTTGCCTTCTTGGGACTG	CAGAATTGCCATTGCACAA
Il1b	GGGCCTCAAAGGAAAGAATC	TACCAGTTGGGGAACTCTGC
Il10	GGTTGCCAAGCCTTATCGGA	TCAGCTTCTCACCCAGGGAA

Table S2. Molecular weights of two polysaccharides

Samples	*Mw* (Da)	*Mn* (Da)	*Mw/Mn* (PI)	Optical rotation, $[\alpha]_{20℃ 589nm}$
BCG-1	4600	4036	1.14	+84.0°
BCG-2	23588	15871	1.49	+145.3°

Table S3. MALDI-TOF MS data of BCG-1.

DP	*Ni*	Mass (experimental)	Mass (theoretical)	Relative error (%)
5	2098	1086.348	1089.531	0.293
6	2171	1292.296	1293.630	0.103
7	1735	1497.863	1497.730	-0.009
8	1538	1703.141	1701.830	-0.077
9	1282	1908.248	1905.930	-0.121
10	1588	2113.066	2110.030	-0.144
11	1168	2317.800	2314.129	-0.158
12	1324	2522.432	2518.229	-0.167
13	1026	2727.036	2722.329	-0.173
14	1100	2931.521	2926.429	-0.174
15	933	3135.750	3130.529	-0.167
16	1037	3340.024	3334.628	-0.162
17	1095	3544.363	3538.728	-0.159
18	882	3748.656	3742.828	-0.155
19	830	3952.973	3946.928	-0.153
20	877	4156.985	4151.028	-0.143
21	1003	4361.225	4355.127	-0.140
22	934	4565.348	4559.227	-0.134
23	1318	4769.514	4763.327	-0.130
24	1014	4973.809	4967.427	-0.128
25	1392	5177.830	5171.527	-0.122
26	953	5382.106	5375.626	-0.120
27	1430	5586.143	5579.726	-0.115
28	1007	5790.399	5783.826	-0.114
29	1596	5994.402	5987.926	-0.108
30	1123	6198.522	6192.026	-0.105
31	1593	6402.586	6396.125	-0.101
32	1019	6606.870	6600.225	-0.101
33	1350	6810.928	6804.325	-0.097
34	793	7015.062	7008.425	-0.095
35	1015	7219.444	7212.525	-0.096
36	568	7423.487	7416.624	-0.092
37	652	7627.689	7620.724	-0.091
38	319	7831.778	7824.824	-0.089
39	361	8035.967	8028.924	-0.088
40	155	8239.989	8233.024	-0.085
41	160	8444.242	8437.123	-0.084
42	60	8648.469	8641.223	-0.084
43	46	8852.579	8845.323	-0.082
44	13	9056.657	9049.423	-0.080
45	13	9260.794	9253.523	-0.079

Table S4. Weight average (*Mw*), number average (*Mn*) and polydispersity index (PI) of BCG-1 determined by HPGPC and MALDI-TOF MS

Methods	*Mw* (Da)	*Mn* (Da)	*Mw/Mn* (PI)
HPGPC	4600	4036	1.14
MALDI-TOF MS	5210	4158	1.25
